# Involvement of ClpE ATPase in Physiology of Streptococcus mutans

**DOI:** 10.1128/Spectrum.01630-21

**Published:** 2021-12-01

**Authors:** Saswati Biswas, Hemendra Pal Singh Dhaked, Andrew Keightley, Indranil Biswas

**Affiliations:** a Department of Microbiology, Molecular Genetics and Immunology, University of Kansas Medical Center, Kansas City, Kansas, USA; b Department of Ophthalmology, University of Missouri School of Medicine, Kansas City, Missouri, USA; Ohio State University

**Keywords:** antibiotics, Clp protease, *Streptococcus mutans*, stress

## Abstract

Streptococcus mutans, a dental pathogen, harbors at least three Clp ATPases (ClpC, ClpE, and ClpX) that form complexes with ClpP protease and participate in regulated proteolysis. Among these, the function of ClpE ATPase is poorly understood. We have utilized an isogenic *clpE*-deficient strain derived from S. mutans UA159 and evaluated the role of ClpE in cellular physiology. We found that loss of ClpE leads to increased susceptibility against thiol stress but not to oxidative and thermal stress. Furthermore, we found that the mutant displays altered tolerance against some antibiotics and altered biofilm formation. We performed a label-free proteomic analysis by comparing the mutant with the wild-type UA159 strain under nonstressed conditions and found that ClpE modulates a relatively limited proteome in the cell compared to the proteomes modulated by ClpX and ClpP. Nevertheless, we found that ClpE deficiency leads to an overabundance of some cell wall synthesis enzymes, ribosomal proteins, and an unknown protease encoded by SMU.2153. Our proteomic data strongly support some of the stress-related phenotypes that we observed. Our study emphasizes the significance of ClpE in the physiology of S. mutans.

**IMPORTANCE** When bacteria encounter environmental stresses, the expression of various proteins collectively known as heat shock proteins is induced. These heat shock proteins are necessary for cell survival specifically under conditions that induce protein denaturation. A subset of heat shock proteins known as the Clp proteolytic complex is required for the degradation of the misfolded proteins in the cell. The Clp proteolytic complex contains an ATPase and a protease. A specific Clp ATPase, ClpE, is uniquely present in Gram-positive bacteria, including streptococci. Here, we have studied the functional role of the ClpE protein in Streptococcus mutans, a dental pathogen. Our results suggest that ClpE is required for survival under certain antibiotic exposure and stress conditions but not others. Our results demonstrate that loss of ClpE leads to a significantly altered cellular proteome, and the analysis of those changes suggests that ClpE’s functions in S. mutans are different from its functions in other Gram-positive bacteria.

## INTRODUCTION

Bacteria are constantly exposed to various environmental stresses, which include fluctuations in nutrients, levels of toxic compounds, oxidative and osmotic stress, and extremes of temperature (1, 2). Under these stress conditions, intracellular proteins become unfolded, which creates a major problem for cell survival. Failure to degrade or refold misfolded proteins results in the formation of insoluble aggregates. Bacteria possess multiple proteases, chaperones, and accessory factors collectively known as heat shock proteins (HSPs) that disaggregate, refold, and/or degrade misfolded proteins and aggregates (3, 4). The process of maintaining proper cellular protein homeostasis is commonly referred to as regulated proteolysis. The caseinolytic protease complex (Clp proteins), or HSP100, is one such group of HSPs that plays a major role in regulated proteolysis. The Clp proteins perform important housekeeping functions, including protein reactivation and remodeling activities typical of molecular chaperones, and target specific proteins for degradation (5, 6). The caseinolytic protease complex consists of two modules: a hexameric complex of Clp ATPases and two heptameric rings of the ClpP protein, which is a serine protease. This Clp complex degrades misfolded proteins that might be toxic for the bacterium under stress-inducing conditions (7). Clp ATPases are highly conserved and ubiquitously distributed proteins and are classified on the basis of the presence of one or two ATP-binding domains and the occurrence of specific signature sequences (8, 9). The class 1 Clp proteins, such as ClpB, ClpC, ClpE, and ClpL, have two ATP-binding domains, whereas the class 2 Clp proteins, such as ClpX, comprise a single domain. Generally, ClpP alone can degrade proteins, but the degradation is not efficient enough to remove the misfolded or unfolded proteins (10). The function of hexameric Clp ATPases is to determine the substrate specificity and then unfold and translocate the substrates into the heptameric ring chamber of ClpP for degradation, where the substrate proteins are cleaved into small peptides (11–14). Some Clp ATPases, such as ClpL and ClpB, do not interact with ClpP; instead, they cooperate with the HSP70 system to disaggregate and refold denatured proteins (15).

Among the three Clp ATPases (ClpC, ClpE, and ClpX) that interact with ClpP to form the proteolytic complex, ClpE is the only one that is strictly found in Gram-positive bacteria (16–18). ClpE is an important member of the HSP100 family and is characterized by the presence of an N-terminal zinc-binding domain (ZBD) whose exact molecular function is currently unknown. However, it has been suggested that ZBD in lactococcal ClpE functions as an oxidative stress sensor (19, 20). ClpE also encodes two AAA+ ATPase domains containing Walker A and B motifs and a tripeptide motif ([I/M/L/V]GF) needed for specific interaction with ClpP (21). The role of ClpE in cellular physiology has not been extensively studied. It has been shown to be involved in various stress tolerance responses, such as thermal, oxidative, and acid stresses (17, 19, 22, 23). Furthermore, ClpE is known to be involved in cell division, biofilm formation, and virulence in many Gram-positive organisms (17, 22, 24, 25). In Bacillus subtilis and other bacteria, ClpE is apparently involved in the degradation of a key heat shock regulator, CtsR, which acts as a transcriptional repressor of key heat shock genes, such as *clpP*, *clpC*, and *clpE* (20, 26).

Streptococcus mutans is a low-GC, Gram-positive oral bacterium associated with dental caries formation in humans. Like other Gram-positive bacteria, S. mutans also encodes a ClpE homolog with a molecular weight of ∼83-kDa (753 amino acids) (Fig. S1 in the supplemental material). The role of ClpE in S. mutans biology is poorly understood, except that it might play a role in biofilm formation (22) and its expression is induced under thermal stress (27). We recently found that ClpE is involved in the degradation of SsrA-tagged proteins in S. mutans (28). SsrA-tagged proteins are predominantly recognized and degraded by ClpX ATPase, but not by ClpC or ClpE ATPases as in other Gram-positive species. Therefore, our finding that ClpE is involved in SsrA tag recognition indicates that this ATPase might be involved in other cellular functions in addition to involvement in the thermal stress response. In this study, we wanted to further explore the functions of the S. mutans ClpE to gain insights into its role in cellular physiology. We found that loss of the ClpE protein leads to differential responses to antibiotics and thiol stresses. In addition, we found that loss of ClpE leads to alterations in the proteome and protein translation.

## RESULTS

### ClpE is required for thiol stress.

**FIG 1:**
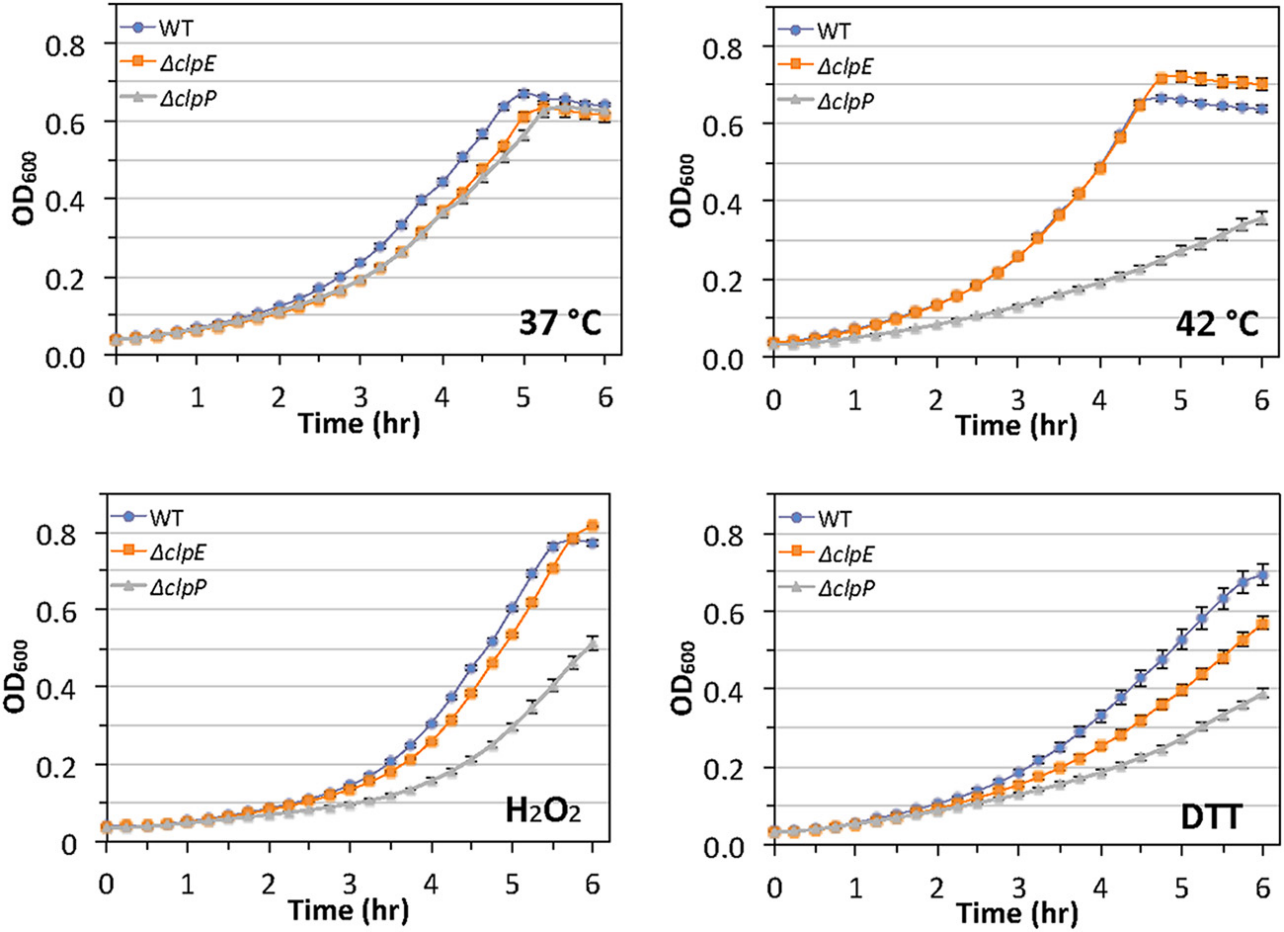
Growth kinetics of UA159 and its isogenic mutants. All strains were grown in BHI medium at 37°C in 96-well microplates, and growth was monitored every 15 min for 6 h using a microplate reader (Tecan Spark). For oxidative and thiol stresses, BHI medium was supplemented with 1 mM hydrogen peroxide or 5 mM DTT, respectively. The effect of high temperature on growth was assessed at 42°C. Growth curves were plotted using three replicates for each strain, and the experiment was repeated three times independently. The data shown are the mean values ± standard deviations from one such experiment. Samples are as follows: WT, UA159 strain; Δ*clpE*, IBSJ5 strain; and Δ*clpP*, IBS512 strain.

To understand the role of the ClpE protein in streptococcal physiology, we first wanted to understand whether ClpE is needed for stress responses in S. mutans. We used the wild-type strain UA159 and its isogenic Δ*clpE* mutant strain IBSJ5, as well as the Δ*clpP* strain IBS512 as a control (28, 29). We first monitored the growth kinetics at 37°C and 42°C for all three strains. As shown by the results in Fig. 1, the Δ*clpE* strain grew equally as well as the UA159 strain at both temperatures, although the rate of growth for both the UA159 and the Δ*clpE* strain was higher at 42°C than at 37°C. As expected, the Δ*clpP* strain showed reduced growth at 42°C. We also noticed that the final biomass was consistently higher for the Δ*clpE* strain than for UA159 when grown at 42°C, indicating that the loss of ClpE might provide selective advantages when thermal shock is the only added stress.

We then tested whether ClpE is required for oxidative or thiol stresses. For this, we used growth medium that was supplemented with either 1 mM H_2_O_2_ or 5 mM dithiothreitol (DTT). These low concentrations of H_2_O_2_ and DTT, which have MICs of ∼0.25×, were used to have less impact on the growth of strain UA159. We found that ClpE was not required for tolerance against oxidative stress, while ClpE was needed for optimum tolerance against thiol stress. However, the effect of lack of ClpE was not as drastic as the effect of lack of ClpP for growth in the presence of DTT. The growth defect of the Δ*clpE* and Δ*clpP* strains was apparent at 1 mM as well (data not shown). When we used 10 mM DTT, the growth for all the strains was drastically reduced; however, both the Δ*clpE* and Δ*clpP* strains grew more slowly than the wild-type UA159 strain (data not shown). When we grew the cultures at 42°C in the presence of 1 mM H_2_O_2_, all of the strains failed to grow (data not shown). However, when we reduced the H_2_O_2_ concentration to 0.5 mM, we observed a growth pattern similar to the pattern we observed with 1 mM H_2_O_2_ at 37°C. In contrast, when the cultures were grown in the presence of DTT at 42°C, the growth kinetics of the Δ*clpE* strain was similar to the growth kinetics of UA159 at 1 mM and 5 mM DTT (data not shown), suggesting that the requirement of ClpE for thiol stress can be alleviated at high temperature.

**FIG 2:**
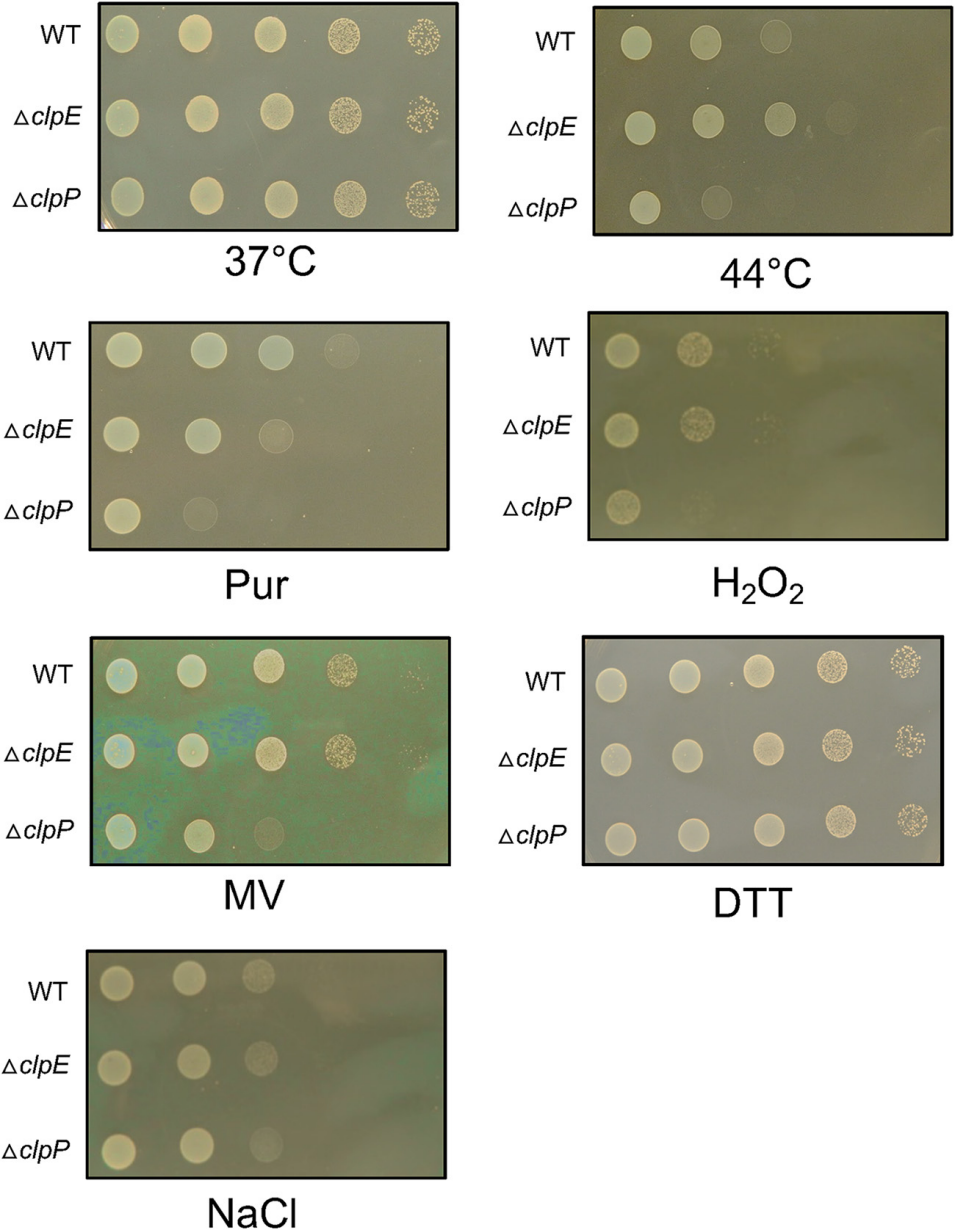
ClpE mutant displays weak phenotype against various stressors on solid medium. BHI agar plates containing various stressor compounds were prepared as indicated. Strains to be tested were first adjusted to similar cell densities (OD_600_ of 2.0) and serially diluted. Aliquots of test strain dilutions (5 μl) were then spotted on BHI agar (control) and BHI agar containing the indicated stressor compound or incubated under the indicated condition (44°C). All plates were incubated under microaerophilic conditions, and all plates except the heat stress plate were incubated at 37°C. Growth was monitored for up to 18 h postinoculation. All the stress tolerance assays were carried out at least twice independently, and data shown are from one such experiment. Stressors are as follows: Pur, puromycin (3 μg/ml); H_2_O_2_ (3 mM); MV, methyl viologen (5 mM); DTT (10 mM); and NaCl (2%).

We also tested the effect of the loss of ClpE on growth on solid medium (agar plate). We found that loss of ClpE had very little effect on growth when the bacteria were exposed to various stressors (Fig. 2), while bacteria with loss of ClpP displayed growth defects when exposed to high temperature (44°C), puromycin (protein synthesis inhibitor), reactive oxygen stress (methyl viologen), oxidative stress (H_2_O_2_), and osmotic stress (NaCl). The only noticeable defect that we observed with the Δ*clpE* strain was against puromycin. We also noticed that all the strains grew equally well when grown on agar plates containing 10 mM DTT (Fig. 2). Taken together, our data suggest that loss of ClpE induces a weak phenotype for cellular growth in the presence of various stressors.

### Loss of ClpE leads to altered antibiotic tolerance.

**FIG 3:**
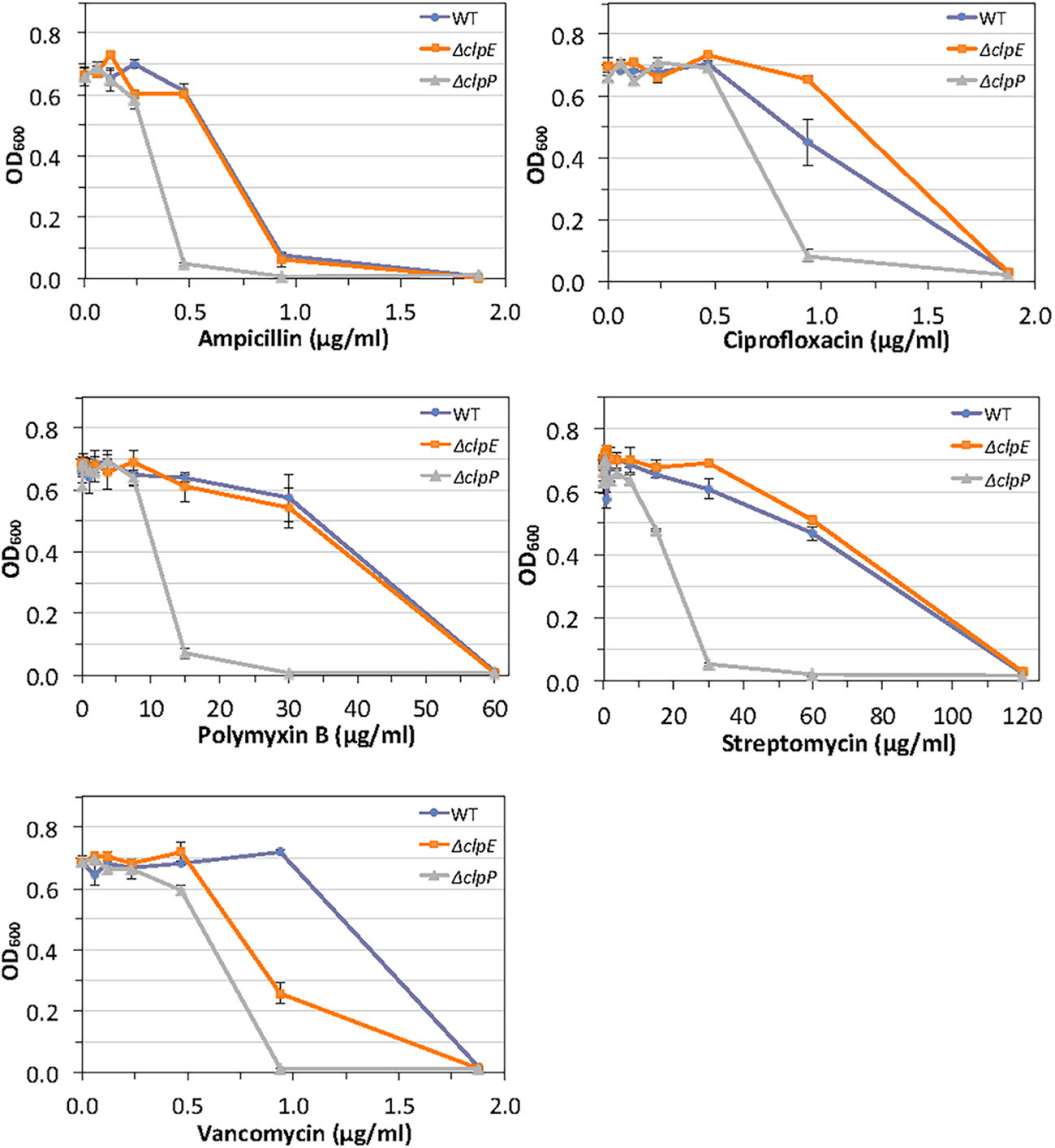
Effects of antibiotics on S. mutans growth. Wild-type UA159 and its isogenic mutant strains were grown under microaerophilic conditions at 37°C in BHI medium containing the indicated antibiotics. After 18 h, cells were mixed gently and the turbidity (OD_600_) was measured using the Tecan Spark. The error bars show the standard deviations of the results from three experiments.

We previously found that ClpP played an important role in antibiotic tolerance (30). To understand whether ClpE is involved in antibiotic tolerance, we used two assays: a plate-based disc diffusion assay and a broth microdilution assay. We included antibiotics that target all of the five known biosynthesis pathways. As shown by the results in Table 1, with the disc diffusion assay, we found that loss of ClpE led to increased sensitivity against chloramphenicol and vancomycin. The difference in the zones of inhibition between the Δ*clpE* and UA159 strains was more pronounced for chloramphenicol (29.0 mm versus ∼27.0 mm) than for vancomycin (18.5 mm versus 17.0 mm). Surprisingly, we also found that the loss of ClpE led to increased tolerance against ciprofloxacin—the zone of inhibition was 20.0 mm for the Δ*clpE* strain versus 24.0 mm for UA159 (Table 1). We also used the Δ*clpP* strain as a control, and as expected, the loss of ClpP led to increased sensitivity toward ampicillin, trimethoprim, and vancomycin (Table 1). The results of the microdilution assay also indicated that loss of ClpE led to decreased sensitivity against ciprofloxacin compared to the sensitivity of the wild-type UA159 strain (Fig. 3). We also found that loss of ClpE led to increased sensitivity toward vancomycin (Fig. 3). However, the MIC values for the Δ*clpE* and UA159 strains were the same (1.8 μg/ml).

**TABLE 1:** Inhibitory zones for various antimicrobials

Antibiotic (amt/disc [μg])	Inhibition zone diam (mm) for indicated strain		
	UA159	Δ*clpE* mutant	Δ*clpP* mutant
Ampicillin (10)	42.0 ± 2.0	42.0 ± 2.0	45.0 ± 2.0*
Chloramphenicol (5)	27.0 ± 1.0	29.0 ± 1.0*	26.0 ± 1.0
Ciprofloxacin (5)	24.0 ± 1.0	20.0 ± 1.0*	24.0 ± 1.0
Fosfomycin (200)	15.0 ± 1.0	15.0 ± 1.0	15.0 ± 1.0
Rifampicin (5)	42.0 ± 2.0	40.0 ± 1.0	40.0 ± 1.0
Trimethoprim (5)	21.0 ± 1.0	21.0 ± 1.0	26.0 ± 1.0*
Vancomycin (5)	17.0 ± 1.0	18.5 ± 1.0*	20.0 ± 1.0*

*, significantly different (*P* < 0.05) from the value for the wild type.

### Loss of ClpE significantly alters the proteome.

To gain an overview of the role of ClpE in protein homeostasis under nonstress conditions, we performed tandem mass tag (TMT)-based quantitative proteomics to compare the proteome profiles of UA159 and the Δ*clpE* strains. We grew the cells to the late-logarithmic growth phase in Todd-Hewitt–yeast extract (THY) broth at 37°C and prepared the samples for TMT analysis as described in Materials and Methods. The S. mutans UA159 strain encodes nearly 2,000 proteins (31); however, under the nutrient-rich growth conditions, we expected that many biosynthetic pathways would be repressed. Nevertheless, we were able to identify nearly 1,200 proteins using a single liquid chromatography-mass spectrometry (LC/MS) gradient run. We found more than 100 proteins that were differentially accumulated (with a cutoff value of 1.2-fold) when ClpE was absent (Table S1). We used this low cutoff value because, previously, we found that for several authentic ClpX protease substrates, the differences in TMT values could be as low as 1.2-fold (32; unpublished data). Surprisingly, we found that loss of ClpE led to the production of at least 12 proteins whose expression levels were increased 2-fold or more. We found that the expression of d-Ala-d-Ala-carboxypeptidase (SMU.75) was increased ∼48-fold compared to its expression in the wild-type strain (Table S1). The function of d-Ala-d-Ala-carboxypeptidase is to remove the C-terminal d-Ala residue from the pentapeptide short chain during cross-linking of peptidoglycan in cell wall biosynthesis. We also found that the expression of d-Ala-d-Ala-ligase was nearly 3-fold higher in the mutant than in the wild-type strain (Table S1). Furthermore, we found that the 30S ribosomal protein S7 and seryl-tRNA synthetase were both more abundant (∼33- and ∼9-fold, respectively) in the mutant than in the wild-type UA159 strain. Another upregulated protein that we found to be overexpressed in the mutant was a putative peptidase, SMU.2153, which was enriched nearly 24-fold in the Δ*clpE* strain compared to its level in the UA159 strain (Table S1).

As for the downregulated proteins, we found only seven proteins whose expression was reduced by 2-fold or more in the mutant Δ*clpE* strain compared to their levels in the wild-type UA159 strain. One notable protein, encoded by SMU.673, was down nearly 30-fold in the mutant. SMU.673 encodes a putative ABC transporter that is highly conserved among streptococci and other related bacteria. We also found that ComYC, a protein needed for the development of competence, was 2-fold reduced in the mutant. The other notable proteins whose expression was reduced were enzymes related to the metabolism of sorbitol and other sugars.

### Loss of ClpE leads to increased protein synthesis.

**FIG 4:**
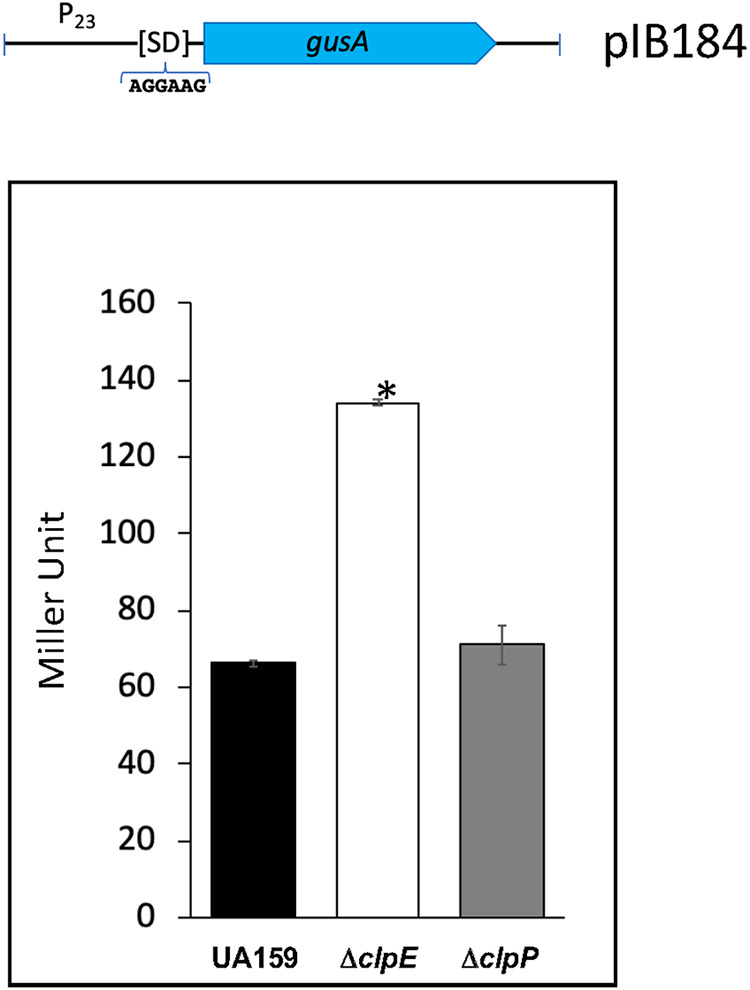
Translation efficiency measurement with a reporter strain. The reporter contains a *gusA* gene that was transcribed from a constitutively expressed promoter (P_23_). The reporter construct is shown at the top. Gus activities were measured in wild-type, Δ*clpE* and Δ*clpP* strains as described in the text. The error bars show the standard deviations of the results from three experiments. An asterisk indicates a significant difference (*P* < 0.05) from the results for the wild type as calculated using the *t* test.

Our proteomic study indicated that the expression of at least two ribosomal proteins (S7 and S20) was increased in ClpE-deficient cells (Table S1). The expression of S7 was increased nearly 30-fold in the Δ*clpE* strain compared to its expression in UA159. Therefore, we decided to measure protein synthesis using GusA as the reporter. We placed the *gusA* gene under a relatively weak ribosome binding site for translation so that we could measure relatively small differences in reporter synthesis. This reporter construct is transcribed from a constitutive promoter so that it is independent of Clp-mediated regulation (Fig. 4, top). We grew the cells containing the reporter and measured the GusA synthesis at mid-log phase at 37°C. As shown by the results in Fig. 4, loss of ClpE led to a nearly 2-fold overexpression of GusA. Taken together, it seems that loss of ClpE led to increased translation in the cell.

### Aberrant cell division observed by SEM and TEM.

**FIG 5:**
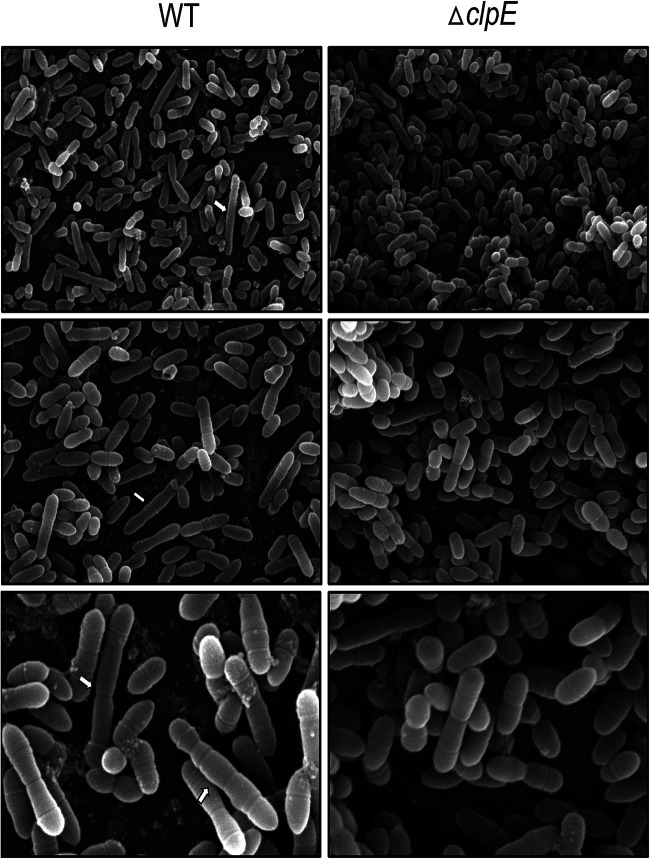
Scanning electron micrograph images of S. mutans strains. The cells were grown on THY agar plates overnight before the samples were processed for SEM. Representative micrographs of each strain were taken at ×2,500 (top), ×8,000 (middle), and ×15,000 (bottom) magnification. Notice that cell shapes are mostly ovococci, which is typical of S. mutans. White arrows indicate longer dividing cells with multiple division septa that are predominantly present in the wild-type UA159 strain (WT). We analyzed three individual fields, and a representative field for each magnification and strain is shown.

Loss of Clp ATPase or ClpP often causes aberrant cell phenotypes (33, 34). We wanted to evaluate whether loss of ClpE results in the generation of any aberrant cell shape or structures. For this, we grew both UA159 and the Δ*clpE* strain on THY agar plates at 37°C for 16 h, collected samples, and immediately processed the cells for both scanning electron microscopy (SEM) and transmission electron microscopy (TEM). We observed that the majority of the cells were ovococcus shaped, which is typical of S. mutans, as reported previously (35, 36). However, we noticed that the UA159 strain also contained numerous elongated cells with multiple division septa (Fig. 5, white arrows). Since we collected our samples from freshly grown streaks from plates, we believe these were cells whose cell divisions were yet to be completed. While these elongated cells were also present in the Δ*clpE* strain, there were many fewer and they were shorter (Fig. 5). Based on the analyses of three individual fields at ×8,000 magnification, we estimated that the number of elongated cells was nearly 5-fold more in the wild type than in the mutant. Furthermore, elongated cells with more than 6 units were only present in the wild-type strain.

When we analyzed the TEM micrographs from both UA159 and the Δ*clpE* strain, none of the elongated cells were visible in the samples even after analyzing multiple fields (data not shown). We also measured the cell wall thickness of UA159 and the Δ*clpE* strain, and we found there was no difference between the strains (Fig. S2).

### Absence of ClpE leads to altered biofilm formation but not bacteriocin secretion.

**FIG 6:**
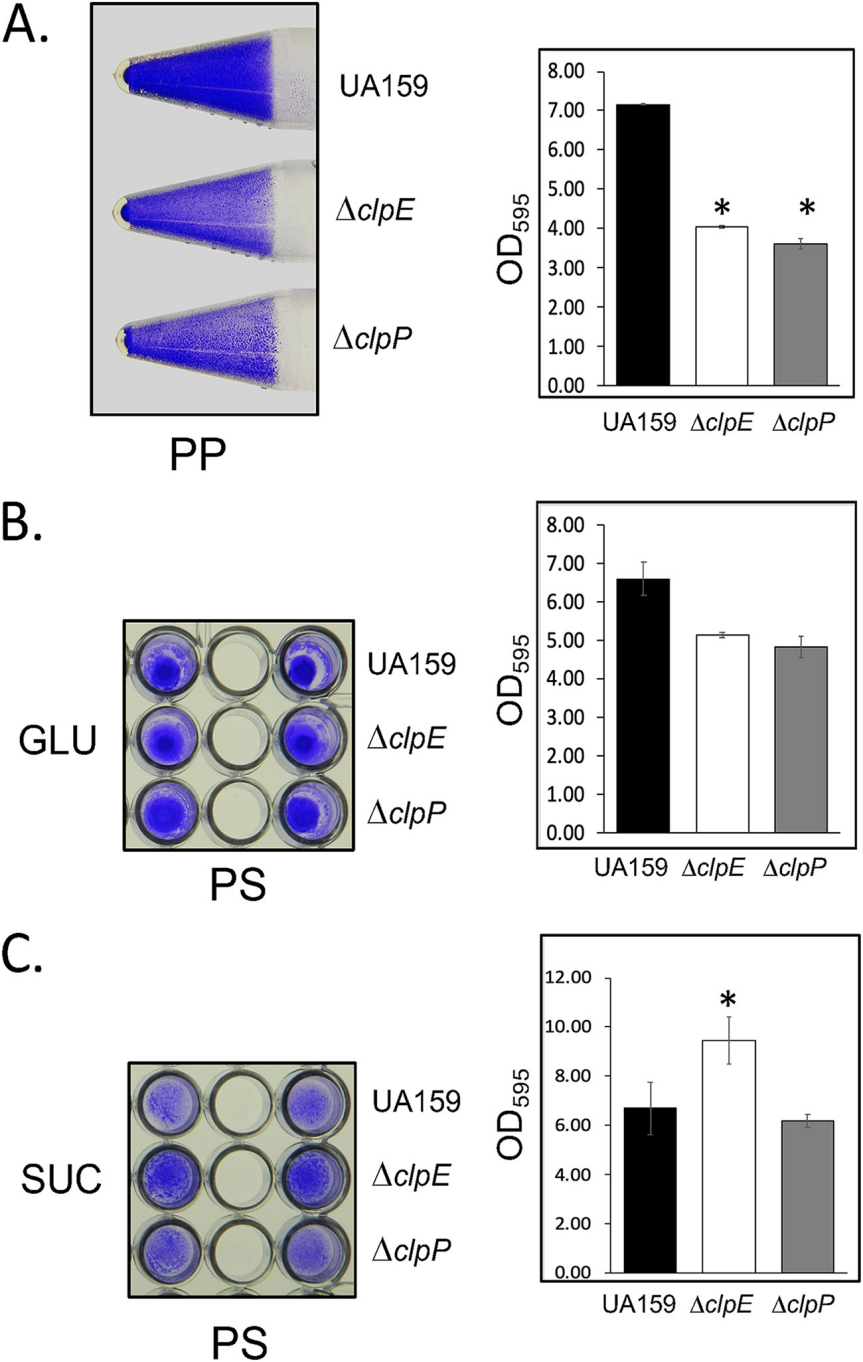
Biofilm assay of S. mutans strains. Cultures of strains to be tested were grown in either 5-ml tubes (A) or 96-well microplates (B and C) to allow biofilm formation on polypropylene and polystyrene, respectively, as inert substrates. All strains were grown in THY supplemented with either 1% sucrose (A and C) or 1% glucose (B) at 37°C for 48 h under microaerophilic conditions to allow biofilm formation. After growth, cultures were aspirated gently, and tubes/wells were washed with PBS to remove planktonic cells, stained with crystal violet, washed to remove unbound dye, and then imaged. Bound dye was later eluted with acetic acid and measured at OD_595_ for quantitation. All biofilm formation assays were performed at least twice independently, and the photographs shown are from one such experiment. PP, polypropylene; PS, polystyrene; GLU, glucose; SUC, sucrose. *, significantly different from the value for the wild type.

We previously found that loss of ClpP led to altered biofilm formation (30). Furthermore, a previous report also suggested that loss of ClpE led to reduced biofilm formation when the mutant was grown on a polystyrene surface (22). Thus, we set up biofilm assays using two types of abiotic surfaces: polypropylene and polystyrene. We used THY medium that was supplemented with either 1% glucose or 1% sucrose. In addition to UA159 and the Δ*clpE* strain, we also included the Δ*clpP* strain as a control. As shown by the results in Fig. 6, we found that loss of both ClpE and ClpP led to decreased sucrose-dependent biofilm formation on a polypropylene surface compared to that of the wild-type strain. On the other hand, we found that when sucrose was absent (1% glucose), there was very poor biofilm formation on a polypropylene surface and no observable differences among the three strains (data not shown). When we used a polystyrene surface, like the polypropylene surface, we observed no differences among the three strains when we used 1% glucose-supplemented medium. In contrast, when we used 1% sucrose-supplemented medium, the Δ*clpE* strain produced nearly 150% more biofilm than the UA159 strain. The amount of biofilm produced by the Δ*clpP* strain was similar to the amount produced by the UA159 strain.

**FIG 7:**
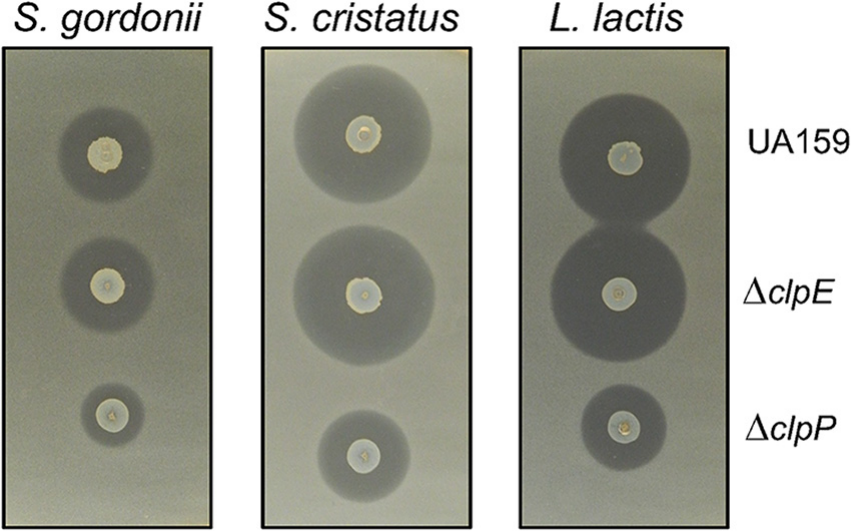
Deferred antagonism assay for mutacin production. Assay was carried out by pouring lawns of the indicator strains of Lactobacillus lactis, Streptococcus gordonii, and Streptococcus cristatus on plates that were spotted with various strains as indicated. Plates were incubated for 8 h after spotting and then overnight after overlay at 37°C under microaerophilic conditions, and images were taken. Experiments were repeated at least twice, and representative plate images are shown.

Since we found that loss of ClpP leads to a decrease in mutacin production in a time-dependent manner (30), we wondered whether ClpE had any effect on mutacin secretion. We assayed for mutacin 8 h after seeding the tester strains by overlaying them with two different streptococcal indicator strains that detect the production of mutacin IV (NlmAB) and one Lactococcus lactis strain that specifically detects production of mutacin V (NlmC). As shown by the results in Fig. 7, there was no noticeable difference in mutacin secretion between the *clpE* mutant and the wild-type strain. As expected, mutacin secretion was reduced in the *clpP* mutant strain. Thus, the effect of ClpP on the secretion of mutacin is independent of ClpE.

### ClpE represses the transcription of *clpP*.

**FIG 8:**
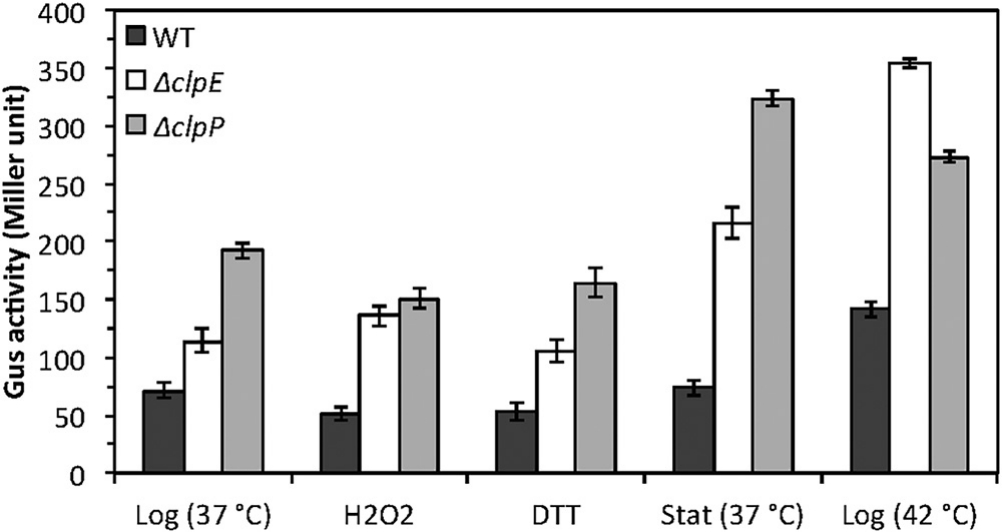
Regulation of P*clpP* by ClpE. Gus activities of P*clpP*-*gusA* reporter strains of wild-type (IBS514), Δ*clpE* (IBST62); and Δ*clpP* (IBST54) were measured under different conditions as described in the text. The error bars show the standard deviations of the results from three experiments. Log, logarithmic phase; Stat, stationary phase.

During an unrelated study, we observed that *clpP* promoter (P*clpP*) activity was modulated by both ClpP and ClpE (37). Therefore, we wanted to study the effect of loss of ClpE on the expression of P*clpP* under various growth conditions, including the presence of stressors. We used previously constructed strains in which a P*clpP-gusA* reporter construct was integrated at an ectopic location (SMU.1405) in the chromosome (37). In addition to the *clpE* mutant strain (IBST62), we also included a *clpP* mutant strain (IBST54) as a control (37) and measured the GusA activity. As shown by the results in Fig. 8, we found that loss of ClpE indeed increased the P*clpP* expression when grown to mid-log phase at 37°C compared to its expression in the wild-type strain. When the cells were grown to stationary phase, we found that P*clpP* expression in the wild-type strain did not change much. In contrast, P*clpP* expression was increased nearly 2-fold in both the *clpE* and *clpP* mutants (Fig. 8), suggesting that these two Clp proteins play a negative role in *clpP* expression. Similarly, we found that when cells were exposed to high temperature (thermal stress), P*clpP* expression was induced in all strains, including the wild-type strain. However, we found that the induction was at its maximum when ClpE was absent (Fig. 8). Loss of ClpP also induced P*clpP* expression, but not as much as in the *clpE* mutant. In contrast, both oxidative stress and thiol stress did not induce P*clpP* expression, suggesting that these two stresses have very little role in P*clpP* induction.

## DISCUSSION

ClpE ATPase is uniquely present in Gram-positive bacteria, including streptococci and other lactic acid bacteria. While ClpE has been studied in B. subtilis, L. lactis, and Listeria monocytogenes, its role in cellular physiology has not been studied in oral streptococci (16–18, 20). Therefore, we decided to evaluate the role of ClpE in the physiology of S. mutans by studying how loss of ClpE modulates a stress tolerance response when cells are exposed to various stress conditions. We also wanted to evaluate how loss of ClpE affects the overall proteome homeostasis. During the study, we made several notable observations that suggest why loss of ClpE results in minor phenotypic defects.

In S. mutans, mutants with loss of ClpE displayed overall weaker phenotypic defects than mutants with loss of ClpX, the major Clp ATPase (38). For example, the *clpE* mutant grew similarly to the wild type when exposed to high temperature or oxidative stress (Fig. 1). However, the mutant was somewhat more sensitive to thiol stress delivered by DTT (Fig. 1). Interestingly, with our plate-based assay, we found that the mutant was slightly more resistant to high temperature but more sensitive to puromycin than the wild type. It has been reported previously that the *clpE* mutant strain of L. monocytogenes grows faster and better than the wild-type strain (17). However, in L. lactis, a *clpE* mutant is more sensitive to puromycin than the wild-type strain (16). Puromycin is a tRNA analogue that prematurely terminates protein synthesis and causes the generation of truncated and misfolded polypeptides that induce a heat shock response in bacteria (39). Our observation that loss of ClpE led to thermotolerance but increased sensitivity to puromycin suggests that ClpE responds differently to these two types of stresses. It is possible that loss of ClpE may increase the expression of some chaperones that help to refold denatured proteins when cells are exposed to higher temperature. In fact, our proteomic study indicates that GroES and GroEL are both increased in the *clpE* mutant strain (Table S1).

The cells lacking ClpE also displayed altered responses when exposed to antibiotics. We found that the *clpE* mutant strain was equally resistant to antibiotics that target the cell membrane (polymyxin B) compared to the wild type. As for the protein synthesis inhibitors, we found that both the *clpE* and wild-type strains were equally resistant to streptomycin (Fig. 3) but the *clpE* strain was more sensitive to chloramphenicol (Table 1). We speculate that the observed differences could be due to the dissimilarity in the modes of action of the antibiotics tested. Streptomycin, an aminoglycoside, binds to the 30S ribosome and interferes with the initiation of protein synthesis. Chloramphenicol binds to the 50S ribosome and inhibits peptidyl transferase activity and peptide bond formation. For cell wall-targeting antibiotics, we found that the *clpE* mutant was equally resistant to ampicillin compared to the wild-type strain but more sensitive to vancomycin (Fig. 3). Ampicillin is a beta-lactam antibiotic with a three-dimensional structure that mimics the d-Ala-d-Ala peptide terminus that serves as the natural substrate for transpeptidase activity of the penicillin-binding protein (PBP) during cell wall synthesis and inhibits the enzyme function. Vancomycin inhibits cell wall synthesis by binding to the d-Ala-d-Ala terminal of the growing peptide chain during cell wall synthesis, resulting in inhibition of the PBP, which prevents further elongation and cross-linking of the peptidoglycan matrix. At present, we are performing additional experiments to understand the reason behind this observation. Surprisingly, we found that loss of ClpE led to greater resistance against ciprofloxacin, a fluoroquinolone antibiotic that inhibits DNA gyrase. We believe this apparent resistance is due to decreased expression of an importer pump. We found that the expression of an ABC transporter type IV encoded by SMU.673 is ∼30-fold more attenuated in the *clpE* mutant strain (Table 1). The SMU.673 protein is a relatively small protein of 265 residues that is highly conserved in S. mutans and contains a conserved motif, COG4905. A COG4905-containing protein, CmpB from Clostridium hathewayi, was shown to be involved with the transport of fluoroquinolones and other drugs (40). The significance of this observation remains to be validated.

We performed our proteomic study under nonstressed conditions because performing proteome analysis under stressed conditions could mask the results due to an overabundance of stress-induced proteins. Surprisingly, we found that the loss of ClpE did not alter the proteome drastically; there were only about 12 proteins whose accumulation was altered ≥2-fold when ClpE was absent (Table S1). Our previous proteomic studies indicated that proteins whose accumulation is altered ∼20% over their base lines in the control are often authentic substrates (30, 32; unpublished data). When we set the cutoff value to 1.2-fold, the total number of proteins with altered accumulation increased to 110 (Table S1). The accumulation of two cell wall biosynthesis enzymes, d-Ala-d-Ala-carboxypeptidase and d-Ala-d-Ala-ligase, was upregulated (47.7- and 3.3-fold, respectively) in the mutant. The other upregulated proteins included the S7 ribosomal protein (32.4-fold) and a tRNA synthetase (9.1-fold). The accumulation of a few other ribosome-associated proteins, such as S20, was also increased (1.4-fold), while L21 was decreased (0.7-fold) in the mutant (Table S1). To verify the significance of the increase of the ribosomal proteins in the mutant, we used a reporter assay for protein translation. We found that loss of ClpE indeed led to an increase in protein synthesis. At present, we are not sure whether the increase in translation is related to a set of specific proteins or overall cellular increase.

Unexpectedly, we also observed that the accumulation of a putative protease encoded by SMU.2135 was increased nearly 23-fold in the mutant (Table 1). SMU.2135 is an ∼48-kDa protein with a COG0612 domain and is highly conserved in S. mutans and other streptococci. It is also predicted to be a Zn-dependent PqqL superfamily of M16-type protease according to the MEROPS database (41). It has been reported that the transcription of SMU.2153 is regulated by autoinducer-2; however, its biological function is currently unknown (42). We constructed SMU.2153-deficient strains in both the wild-type and the *clpE* background, and we did not observe any noticeable phenotypic changes when the cells lacked SMU.2135. Thus, the biological role of S. mutans SMU.2135 remains unclear.

Two other notable overexpressed proteins in the mutant are SMU.917 and SMU.1527. The first encodes a putative 6-pyruvoyl tetrahydropterin synthase, and the latter encodes the epsilon subunit of the F_0_/F_1_ ATPase. While the exact significance of this observation is under investigation, we believe that SMU.917, which is a homolog of Escherichia coli QueD, might be involved in the synthesis of cofactors that are necessary for growth, as it is in other bacteria (43, 44).

Loss of ClpE did not produce drastic changes in the cellular morphology, except that longer dividing cells were fewer in the mutant than in the wild type as observed by SEM (Fig. 5). However, a closer analysis by TEM indicated that ClpE deficiency led to asymmetric cell division (Fig. 6). Since we observed increased expression of d-Ala-d-Ala-carboxypeptidase in the mutant and since d-Ala-d-Ala-carboxypeptidases are known to alter cell shape, we speculate that the observed asymmetric cell septum formation is due to altered d-Ala-d-Ala-carboxypeptidase expression (45). However, upon closer analysis of the TEM micrograph, we did not find any change in the cell wall thickness (Fig. S2). It is important to mention that loss of ClpE in L. monocytogenes does not alter the cellular morphology as analyzed by TEM (17). Altered morphology is only apparent when cells are lacking both ClpC and ClpE (17).

ClpE forms a complex with ClpP for the degradation of misfolded proteins during stress responses. The transcription of *clpP* and a few other genes, including *clpE*, is repressed by CtsR in bacteria (14, 46). The regulation of *clpP* in S. mutans is unique, since the transcription is also activated by some unknown regulator in addition to CtsR repression (37, 47). Our previous study has suggested that ClpE is involved in the repression of *clpP* transcription (37). Expectedly, we found that loss of ClpE leads to increased *clpP* transcription as measured by reporter assay (Fig. 8). Similarly, ClpP also autoregulates its own expression. Surprisingly, we found that *clpP* transcription is derepressed more when cells are in the stationary phase than when they are in the logarithmic phase in the absence of either ClpP or ClpE (Fig. 8). This suggests that other repressors in addition to CtsR might be involved in the modulation of *clpP* transcription. In B. subtilis, the cellular CtsR level is primarily controlled by the ClpC-ClpP complex during thermal and thiol stresses (19, 48, 49). However, ClpE is also indicated to be involved with CtsR degradation in B. subtilis and L. lactis, by less understood processes (20, 26). In contrast, our previous study demonstrates that ClpE does not play a role in CtsR degradation in S. mutans (50). Thus, we believe that there is the presence of another regulator that is either directly or indirectly modulated by ClpE in S. mutans. This could also explain why the level of *clpP* transcription was higher in the *clpE* mutant than in the *clpP* mutant.

In B. subtilis, there are approximately 1,400 ClpX hexamers, 250 ClpC hexamers, and only 100 ClpE hexamers per cell during the logarithmic-growth phase (51). After thermal shock, while the number of hexamers of ClpX remains the same, the number of ClpE hexamers increases nearly 4.5-fold (51). Thus, ClpE probably is needed for alleviating the load of misfolded proteins after thermal shock. However, our results indicated that loss of ClpE led to increased survival of the cells at higher temperature in S. mutans. While the exact role of ClpE in the cellular physiology remains unclear, the proteomic data generated in this study provide some clues about its role in various cellular processes. Further proteomic studies performed under various stress conditions are necessary to obtain a clearer picture.

## MATERIALS AND METHODS

### Bacterial strains and plasmids.

S. mutans strain UA159 was used as the wild type, and its isogenic Δ*clpE* (strain IBSJ5) and Δ*clpP* (strain IBS512) mutants are described elsewhere (28, 29). Cells were routinely grown in Todd-Hewitt medium (Becton, Dickinson) containing 0.2% yeast extract (THY) at 37°C under microaerophilic conditions (in a candle jar or statically) and in the presence of appropriate antibiotics, such as erythromycin (5 μg/ml), when needed. For some experiments, Bacto brain heart infusion (BHI; Becton, Dickinson) broth was also used. For growth kinetics assays, the effects of dithiothreitol (DTT; reducing agent), hydrogen peroxide (H_2_O_2_; oxidizing agent), and high temperature (42°C) on growth were also measured. Cells from overnight cultures were diluted in BHI medium to adjust the initial optical density at 600 nm (OD_600_) to 0.05. Cells were then grown in the presence or absence of 1 mM H_2_O_2_ or 5 mM DTT at 37°C in a 96-well plate covered with a lid. The OD_600_ was measured at 15-min intervals for 6 h using a microplate reader (Tecan Spark). In a different set, cells were also grown at 42°C to study the effect of high temperature on growth. Experiments were repeated three times or more.

### Antibiotic susceptibility assays.

The susceptibility of the S. mutans strains to ampicillin, ciprofloxacin, polymyxin B, streptomycin, and vancomycin was monitored using the microdilution method, following Clinical and Laboratory Standards Institute (CLSI) guidelines with slight modifications (37). We used a 96-well plate with a final volume of 200 μl per well. All the wells (A1 to A12, for example) were first filled with 150 μl of BHI medium. The concentration of each antibiotic was made (in BHI medium) two times higher than required in the first well in BHI medium. An amount of 150 μl from 2× stocks was added to the first well (A1), which diluted the antibiotic to 1×. Then, 150 μl from A1 was added to A2 and mixed in, and this serial dilution continued up to A11. The last well, A12, was used as a growth control with no antibiotic. Independently, cells were grown in BHI medium overnight at 37°C. Cells were diluted in BHI medium to adjust the OD_600_ to ∼0.1. An amount of 50 μl from this diluted culture was added into each well and mixed gently, which reduced the final concentrations of antibiotics by 25% in each well. Cells were further incubated at 37°C under microaerophilic conditions (in a candle jar). After 18 h, cells were mixed gently, and the OD_600_ was measured using a Tecan Spark instrument. The OD_600_ of BHI medium was subtracted as a blank. The OD_600_ values were plotted against the concentrations of the antibiotics, and the MIC values were noted.

Disc diffusion assays were also performed to evaluate the antibiotic susceptibility of S. mutans UA159 and its derivatives as described previously (52). Briefly, three or four freshly grown bacterial colonies from a THY agar plate were resuspended in 0.85% NaCl and the initial optical density (OD_600_) was adjusted to 0.1. Cultures were spread onto BHI plates with a cotton swab. Antibiotic disks (6 mm in diameter; Becton and Dickinson Laboratories) were then placed on the inoculated plates, and the plates incubated for 16 h. For each antibiotic, two discs were used, and the experiments were repeated twice on different days. The zones of inhibition were measured after overnight incubation at 37°C under microaerophilic conditions. The diameters of the zones of inhibition were noted, and the *t* test was performed to determine statistical significance of differences.

### Stress susceptibility assays.

The sensitivity of various S. mutans strains to different stressors was measured as described previously, with some modifications (52–54). Briefly, cells were grown from a 1:20 dilution in BHI overnight at 37°C. Cells were harvested and resuspended in phosphate-buffered saline (PBS). The initial culture density was adjusted to an OD_600_ of 2.0 (∼5 × 10^9^ cells/ml) for each strain. Cell suspensions were then serially diluted up to 10^−4^. Five-microliter amounts of diluted culture were spotted on BHI agar plates containing 10 mM DTT, 5 mM methyl viologen (MV; oxidizing agent), 3 mM hydrogen peroxide, 3 μg/ml puromycin (thermal stress mimic), or 2% NaCl (osmotic stress). Plates were then incubated for 18 h at 37°C under microaerophilic conditions before images were taken. In a different set, plates were incubated at 44°C for 18 h and then photographed.

### Gus assay.

β-Glucuronidase (Gus) assays of IBS514 (UA159), IBST62 (Δ*clpE*), and IBST54 (Δ*clpP*) strains were performed under stress conditions to check their ability to activate the P*clpP-gusA* reporter fusion and to measure protein translation using a plasmid containing the *gusA* gene under the constitutive promoter P_23_ (55). Cells were grown from a 1:20 dilution in BHI (or THY) medium at 37°C until the OD_600_ reached 0.4 to 0.6, and the values were noted. Three-milliliter amounts of cultures were harvested, washed with PBS, and stored at −20°C. Each pellet was resuspended in 760 μl of Z buffer (60 mM Na_2_HPO_4_, 40 mM NaH_2_PO_4_, 10 mM KCl, 1 mM MgSO_4_, 20 mM DTT), 40 μl of freshly prepared 10-mg/ml lysozyme, and 8 μl of 10% Triton X-100. The reaction mixture was incubated further at 37°C for 30 min. Two hundred microliters of *p*-nitrophenyl-d-glucoside (PNPG) (4 mg/ml in Z buffer) was added to the reaction mixture, and the mixture incubated at 37°C for 20 min. After a yellow color had developed, 400 μl of 1 M Na_2_CO_3_ was added to the reaction mixture to stop the reaction and the time was noted. The absorbance of the supernatant at 420 nm was measured. Gus activity was calculated as (1,000 × OD_420_)/(time in min × OD_600_) in Miller units (37).

### Biofilm formation assay.

The biofilm formation assay was done in 96-well U-bottom polystyrene plates and in 5-ml polypropylene tubes. THY broth containing either 1% sucrose or 1% glucose was used for the biofilm formation as described previously (30, 56). Briefly, for the 96-well format, 290 μl medium was inoculated with 10 μl culture that was adjusted to an OD_600_ of 0.2. Plates were incubated at 37°C under microaerophilic conditions (in a candle jar) for 72 h. The planktonic cells were removed by flooding the plate with water. Plates were then dried and incubated for 60 min at 55°C to fix the cells. Biofilms were stained with 0.1% crystal violet stain for 30 min. The stain was discarded, and the plates were washed thrice with water and dried at 37°C before imaging. Afterwards, the stain was eluted with 300 μl of 33% glacial acetic acid and the intensity of the adhered stain was measured at OD_595_. For polypropylene tubes, 3 ml THY broth containing 1% sucrose was inoculated with 50 μl of culture adjusted to an OD_600_ of 0.2 and incubated at 37°C for 72 h. The planktonic cells were removed by decanting, and the tubes washed with water by dipping them. The tubes were dried, and the adhered biofilm cells were fixed for 60 min at 55°C. Cells were stained with crystal violet, washed again, dried, and subjected to imaging. The adhered stain was eluted and measured as described above.

### Deferred antagonism assay.

Mutacin production by S. mutans was evaluated by a deferred antagonism assay (30). Briefly, S. mutans UA159 and its derivatives were grown overnight in THY broth, harvested, and resuspended in PBS. The initial culture density was adjusted to an OD_600_ of 2.0 (∼5 × 10^9^ cells/ml) for each strain. Samples (4 μl) were spotted on THY agar plates and incubated for 8 h at 37°C in a candle jar. The plates were then overlaid with 5 ml of soft agar containing freshly grown overnight cultures (400 μl) of the indicator strains of choice (Streptococcus gordonii strain DL-1, Streptococcus cristatus strain 5100, and Lactococcus lactis strain MG1363). The plates were allowed to solidify at room temperature for 10 min. Then, they were incubated overnight at 37°C under microaerophilic conditions. The following day, the zones of inhibition of the indicator strains were evaluated for the different S. mutans strains.

### Quantitative proteomic analysis.

Cultures grown overnight were diluted in THY (1:20) and incubated at 37°C until the OD_600_ reached 1.0. Cells were then harvested and washed with PBS. Cell pellets were then resuspended in B-PER solution (Thermo Scientific) and lysed using a bead beater. The lysate was centrifuged to remove the cell debris and stored at −20°C until further use. Total protein extracts were reduced, alkylated, and subjected to proteolytic digestion using filter-aided sample preparation (FASP) essentially as described previously (52). About 100 μg protein was processed for each sample. After overnight digestion, the peptidomes were harvested by centrifugation, dried down, and resuspended in pure water.

For tandem mass tag (TMT) quantitation, peptidomes were labeled with a TMT reagent (Thermo Scientific) and then purified by solid-phase extraction (SPE) using Isolute C_18_(EC) spin columns (BioTage). An equal quantity of each sample was mixed for multidimensional protein identification technology (MudPIT), and offline fractionation (1st dimension) was carried out on an XBridge peptide BEH130 C_18_ column (100 mm by 400 μm; Waters) under basic conditions.

Peptides were fractionated in a single, two-step reversed-phase gradient of buffer A (10 mM ammonium formate, ∼pH 10) and buffer B (acetonitrile with 5% buffer A) as follows: 1% to 30% buffer B from 0 to 100 min, 30% to 40% buffer B from 100 to 120 min, at a constant flow rate of 8 μl/minute. Twenty-four 5-min fractions were pooled into 8 samples (every 8th fraction) and dried down for LC-MS. The 8 MudPIT fractions were further resolved by acidic reversed-phase LC-MS with data-dependent acquisition (QExactive plus MS system), acquiring MS survey scans at 70,000 resolution and 18 dependent scans per cycle at 17,500 resolution. Data files from MudPIT were merged and searched with Mascot version 2.6 (Matrix Science) against the S. mutans UA159 protein database (4,840 sequences) with a reversed-sequence decoy database search, applying a significance threshold of *P* < 0.05. For protein identification, the peptide mass tolerance was 8 ppm and the tandem MS (MS/MS) peak tolerance was 0.02 Da, allowing 1 missed cleavage for identification. TMT ratios were normalized by using the average ratio of all peptides.

### SEM and TEM.

Cultures of S. mutans UA159 and the Δ*clpE* strain were streaked on THY agar plates and incubated at 37°C under microaerophilic conditions. Cells were collected from agar plates and resuspended in PBS. Cells were washed twice in PBS and then fixed (2.0% glutaraldehyde in 0.1 M sodium cacodylate buffer) for 24 h. In another set, fresh overnight cultures were also grown in THY overnight at 37°C and cells were harvested. Cells were washed in PBS and then fixed (2.0% glutaraldehyde in 0.1 M sodium cacodylate buffer) for 24 h. For TEM, samples were further treated in 1% osmium tetroxide for 1 h for postfixing, followed by dehydration in a graded series of ethanol concentrations (50%, 70%, 80%, 95%, and 100%). Dehydration of samples with 100% ethanol was followed by 2 exchanges of propylene oxide and then infiltration with 50:50 propylene oxide/Embed 812 resin overnight. The next day, samples were embedded into 100% Embed 812 molds and polymerized at 60°C. Polymerized blocks were trimmed and sectioned with a diamond histo-knife, and 1-μm sections were stained with toluidine blue. The best blocks were sectioned with a diamond knife in a Leica EM UC7 ultramicrotome. Thin (75-nm) sections were placed on copper grids. The grids were treated for contrast with 3% uranyl acetate for 5 min, followed by 3% Reynold’s lead citrate for 5 min, and then viewed using a JEOL JEM-1400 TEM at 100 kV. Digital images were acquired with an AMT digital camera.

For SEM, samples were collected from the agar plates and fixed with glutaraldehyde as described above. Postfixation was carried out in 1% osmium tetroxide, followed by dehydration in a graded series of ethanol concentrations. Samples were then treated with a graded series of hexamethyl disilazane (HDMS) concentrations, and the cell pellets were air dried overnight. Samples were then mounted, sputter coated with gold/palladium (EMS/Quorum 150 R ES sputter coater), and viewed in a Hitachi S-2700 SEM. Images were captured as TIFF files with the Quartz PCI digital capture system. We analyzed three individual fields for each stain at ×8,000 magnification to determine the number of elongated cells present in the field.
