# Supplementary material for: Involvement of ClpE ATPase in Physiology of Streptococcus mutans
Source: Microbiol Spectr. 2021 Dec 1;9(3):e01630-21. doi: 10.1128/Spectrum.01630-21 (PMC8635124; doi:10.1128/Spectrum.01630-21)
Supplement: SUPPLEMENTAL FILE 1 — Supplemental material. Download SPECTRUM01630-21_Supp_1_seq12.pdf, PDF file, 1.1 MB [file spectrum01630-21_supp_1_seq12.pdf]

Table: S1. Differentially expressed proteins in Δ*clpE* strain

Accession #	Description	**Score**	Δ*clpE*/WT	MS2 scans	peptides	emPAI
SMU_75	D-ala-carboxypeptidase	289	47.755	10	4	0.44
SMU_358	30S ribosomal protein S7	1697	32.469	52	5	2.77
SMU_2153c	Putative peptidase	79	23.527	5	5	0.28
SMU_1527	ATP synthase F1	128	22.487	3	1	0.18
SMU_917c	Tetrahydropterin synthase	287	18.836	10	3	0.55
SMU_1886	Seryl-tRNA synthetase	1180	9.195	36	7	0.74
SMU_368c	Hypothetical protein	169	3.993	13	5	0.3
SMU_1568	Sugar-bind protein MalX	1269	3.56	53	8	0.71
SMU_603	D-alanyl-D-alanyl ligase	34	3.37	1	1	0.05
SMU_684	HNH/ENDO VII family nuclease	511	2.256	25	8	1.14
SMU_2038	PTS system, IIABC component	607	2.074	22	5	0.22
SMU_46	Hypothetical protein	38	2.034	1	1	0.11
SMU_44	Conserved hypothetical protein	17	1.825	1	1	0.12
SMU_1947	Antitermination factor	757	1.599	42	4	1.5
SMU_1063	Putative ABC transporter	482	1.568	21	6	0.42
SMU_1672	ATP-dependent Clp protease	157	1.564	14	4	0.6
SMU_1674	Putative aminotransferase	47	1.449	1	1	0.06
SMU_1978	Acetate kinase	1131	1.436	51	9	1.04
SMU_1403c	Endonuclease Cas2	54	1.435	1	1	0.22
SMU_1405c	Conserved hypothetical protein	1028	1.425	52	23	0.45
SMU_1127	Putative 30S protein S20	720	1.424	32	3	1.5
SMU_274	Hexulose-6-phos isomerase	111	1.405	4	1	0.08
SMU_233	Ketol-acid reductoisomerase	2252	1.354	89	14	2.52
SMU_992	Hypothetical protein	17	1.343	1	1	0.07
SMU_1954	Chaperonin GroEL	6675	1.331	245	19	2.22
SMU_1955	Co-chaperonin GroES	1327	1.329	37	3	2.31
SMU_1461	dTDP-glucose synthase	876	1.326	34	10	2.21
SMU_1207	Fic family protein	40	1.322	1	1	0.12
SMU_2147c	LysM PBD containing protein	88	1.321	4	3	0.31
SMU_1996	Isopentenyl kinase	33	1.319	2	2	0.17
SMU_373	Methyltransferase protein	37	1.314	2	1	0.09
SMU_2074	NTP reductase	1027	1.297	45	12	0.6
SMU_997	ABC transporter	67	1.271	5	3	0.28
SMU_218	Transcriptional regulator	22	1.259	1	1	0.19
SMU_1160c	Hypothetical protein	54	1.246	2	2	0.14
SMU_1639	Methionyl-tRNA synthetase	868	1.243	42	10	0.42
SMU_669c	Putative glutaredoxin	45	1.238	3	1	0.3
SMU_816	Putative aminotransferase	110	1.222	7	4	0.28
SMU_1999c	γ-glutamylcysteine synthetase	93	1.216	5	2	0.11
SMU_73	PFL family protein	273	1.212	17	4	0.25
SMU_873	Homocys. methyltransferase	64	1.21	4	4	0.13
SMU_209c	ydcP family protein	97	1.207	5	3	0.58
SMU_1296	Glutathione S-transferase	675	1.202	28	7	1.41
SMU_673	ABC transporter permease	20	0.033	3	1	0.18
SMU_1423	TPP-dependent E1	131	0.407	12	5	0.49
SMU_1490	Beta-galactosidase	416	0.415	23	9	0.63
SMU_1493	Tagatose-aldolase	267	0.416	12	4	0.52
SMU_1494	Phosphotagatokinase	71	0.482	2	1	0.08
SMU_310	Sorbitol operon activator	16	0.485	1	1	0.14
SMU_1491	EIICB-Lac	344	0.498	17	5	0.25
SMU_1984	Competence protein ComYC	42	0.50	2	2	0.49
SMU_272	PTS system, EIIA component	48	0.539	1	1	0.16
SMU_312	PTS sorbitol IIBC	169	0.568	5	2	0.16

SMU_1005	Glucosyltransferase-SI	5903	0.572	260	36	1.45
SMU_308	Sorbitol-dehydrogenase	195	0.595	7	2	0.2
SMU_1004	Glucosyltransferase-I	5730	0.612	236	35	1.38
SMU_609	40K cell wall precursor	536	0.627	27	7	0.31
SMU_689	GBS Bsp-like protein	758	0.64	43	17	0.67
SMU_1632	MTA/SAH nucleosidase	104	0.651	2	1	0.11
SMU_1967	SSB protein	47	0.652	1	1	0.19
SMU_469	Putative recombination prot U	31	0.667	1	1	0.12
SMU_2086	CinA	266	0.677	9	2	0.19
SMU_846	50S ribosomal protein L21	99	0.688	2	1	0.21
SMU_89c	Putative nitrite transporter	21	0.699	1	1	0.09
SMU_2112	Glucan-binding GbpA	394	0.703	16	7	0.34
SMU_401c	GNAT family N-acetyltrans.	95	0.703	2	2	0.35
SMU_1723c	HD domain-containing protein	51	0.708	3	1	0.13
SMU_13	Putative cell-cycle protein	15	0.708	1	1	0.05
SMU.1601	Glucosidase	99	0.71	6	4	0.21
SMU_1709	Potassium uptake protein TrkH	16	0.714	1	1	0.05
SMU_116	Tagatose 1,6-aldolase	28	0.721	1	1	0.07
SMU_1537	GlgD	420	0.722	21	6	0.66
SMU_755	Diacylglycerol transferase	20	0.724	1	1	0.1
SMU_127	Acetoin dehydrogenase	161	0.725	7	4	0.34
SMU_967	Folyl-polyglutamate synthetase	24	0.728	1	1	0.06
SMU_470	DUF1273 containing protein	19	0.736	1	1	0.13
SMU_1227	Purine phosphorylase	53	0.738	2	1	0.11
SMU_910	Glucosyltransferase-S	463	0.739	31	14	0.29
SMU_591c	Hypothetical protein	28	0.745	3	1	0.06
SMU_1717c	NTP diphosphatase	40	0.751	3	3	0.24
SMU_814	MutT-like protein	25	0.752	1	1	0.16
SMU_2067	Stress response protein	60	0.753	4	2	0.15
SMU_533	Anthranilate synthase	22	0.753	1	1	0.14
SMU_1418	Coproporphyrinogen III oxidase	17	0.759	1	1	0.06
SMU_695	LysM PBD-containing protein	323	0.76	13	2	0.35
SMU_1904c	Thiol reductase thioredoxin	88	0.762	5	3	0.23
SMU_1517	VicR-like protein	298	0.765	18	4	0.48
SMU_906	ABC transporter permease	76	0.765	6	3	0.13
SMU_1299c	Putative acetate kinase	44	0.767	2	1	0.22
SMU_497c	YigZ family protein	14	0.768	1	1	0.11
SMU_427	Copper chaperone	151	0.771	5	2	1.28
SMU_275	Ribulose-epimerase	144	0.773	7	4	0.64
SMU_1780	Recombination regulator RecX	40	0.775	2	1	0.08
SMU_341	Putative deoxyribonuclease	65	0.775	3	3	0.27
SMU_1536	Glycogen synthase	244	0.779	12	4	0.22
SMU_1707c	Putative rRNA methylase	31	0.779	1	1	0.13
SMU_426	Cu-trans ATPase	144	0.779	8	4	0.14
SMU_140	Putative glutathione reductase	20	0.781	2	2	0.12
SMU_962	Putative dehydrogenase	109	0.782	4	3	0.23
SMU_882	ATP-binding protein MsmK	758	0.783	43	9	0.99
SMU_237c	Membrane protein	214	0.783	10	4	0.33
SMU_826	Rhamnosyltransferase	68	0.784	2	1	0.07
SMU_2057c	P-type ATPase	231	0.785	12	5	0.22
SMU_538	Tryptophan synthase	37	0.789	4	1	0.1
SMU_415	Phosphotrans. family protein	179	0.789	6	2	0.29
SMU_1622	Sulfoxide reductase	76	0.79	5	2	0.32
SMU_1347c	ABC transporter subunit MubY	711	0.792	41	9	0.46
SMU_1201c	DUF2969 containing protein	101	0.793	3	2	1.21
SMU_1197	TVP38/TMEM64 family protein	15	0.796	1	1	0.11
SMU_337	Putative membrane protein	76	0.797	3	3	0.28
SMU_1218	Putative amidase	38	0.797	3	3	0.17

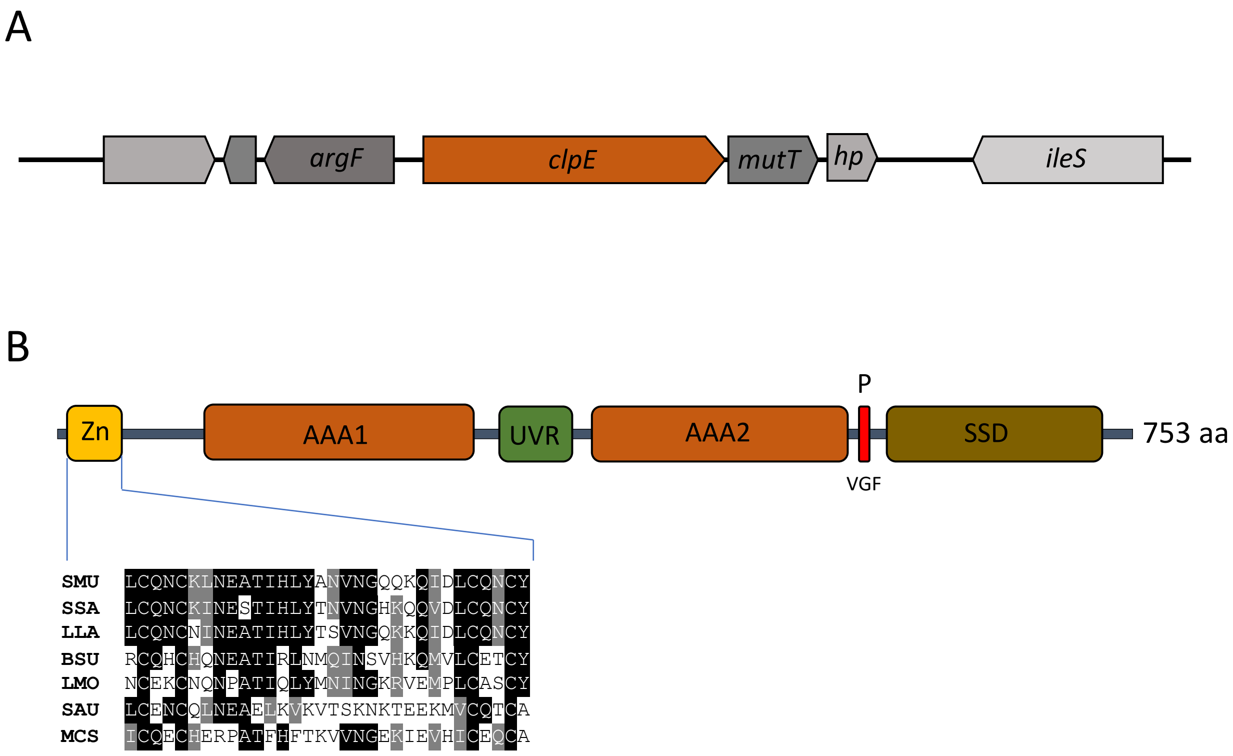

Figure S1. ClpE ATPase of *S. mutans.* A) Genetic organization of the *clpE* locus in *S. mutans.* The nearby genes are not conserved in all streptococci, although the downstream genes, *mutT* and *hp* are topologically linked and are present near the *clpE* locus in streptococci. B) Schematic diagram of ClpE protein with various domains. ClpE encodes two AAA+ ATPase domains separated by a small helical domain that shows homology with UVR proteins. A ClpP interacting motif with VGF residues are shown. The N-terminal region contains a highly conserved zinc binding domain that is also present in McsA protein.

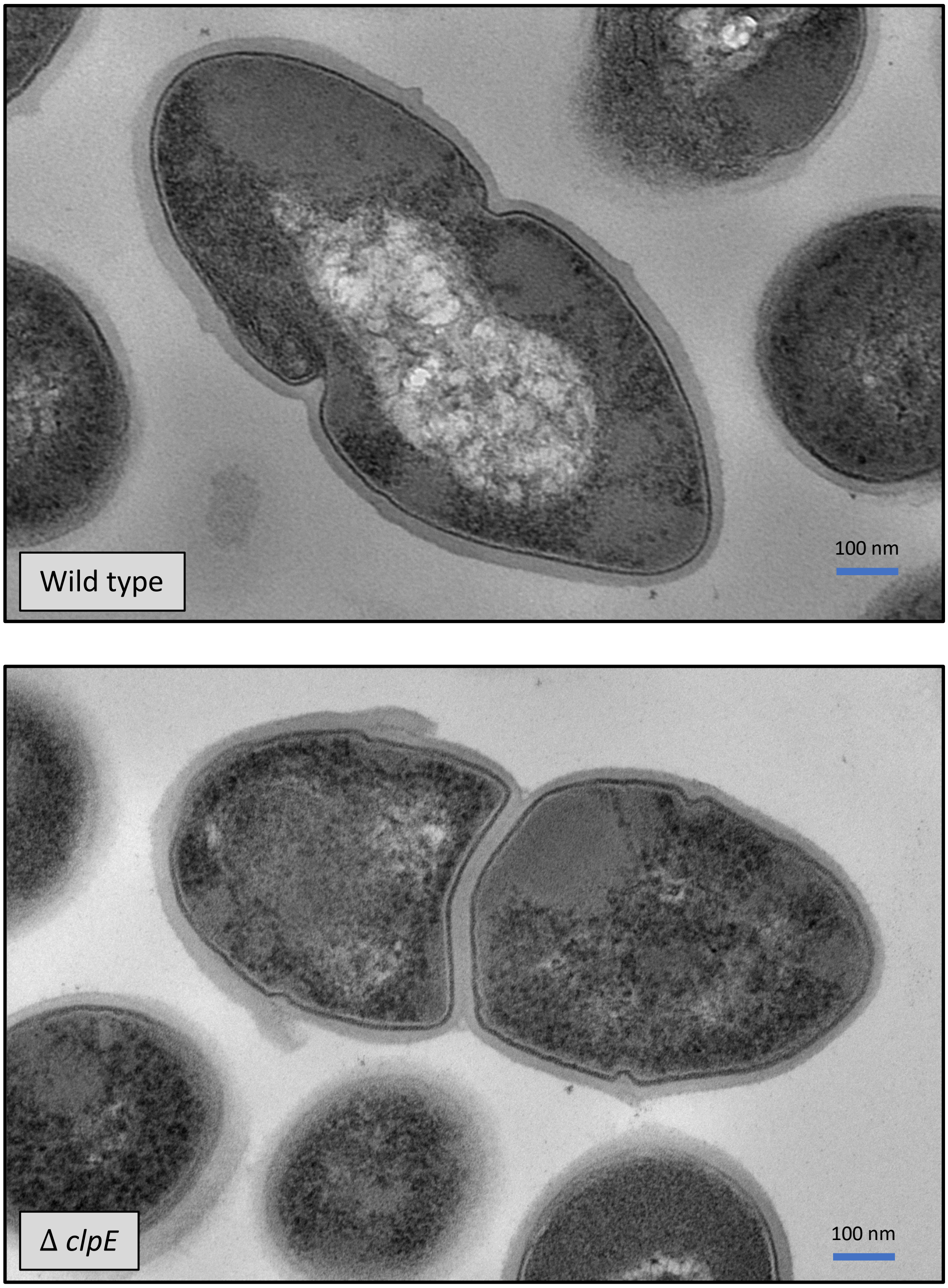

Figure S2. Transmission electron micrographs of *S.mutans* cells grown at 37ºC. Note, the thickness of the cell-wall is very similar to both the wild-type and the *clpE* mutant.
